# Estimation of User-Applied Isometric Force/Torque Using Upper Extremity Force Myography

**DOI:** 10.3389/frobt.2019.00120

**Published:** 2019-11-22

**Authors:** Maram Sakr, Xianta Jiang, Carlo Menon

**Affiliations:** Menrva Research Group, Schools of Mechatronic Systems and Engineering Science, Simon Fraser University, Burnaby, BC, Canada

**Keywords:** hand force/torque estimation, human-machine interaction, force myography, wearable sensors, multi-output regression

## Abstract

Hand force estimation is critical for applications that involve physical human-machine interactions for force monitoring and machine control. Force Myography (FMG) is a potential technique to be used for estimating hand force/torque. The FMG signals reflect the volumetric changes in the arm muscles due to muscle contraction or expansion. This paper investigates the feasibility of employing force-sensing resistors (FSRs) worn on the arm to measure the FMG signals for isometric force/torque estimation. Nine participants were recruited in this study and were asked to exert isometric force along three perpendicular axes, torque about the same three axes, and force and torque simultaneously. During the tests, the isometric force and torque were measured using a 6-degree-of-freedom (DoF) (i.e., force in three axes and torque around the same axes) load cell for ground truth labels whereas the FMG signals were recorded using a total number of 60 FSRs, which were embedded into four bands worn on the different locations of the arm. A two-stage regression strategy was employed to enhance the performance of the FMG bands, where three regression algorithms including general regression neural network (GRNN), support vector regression (SVR), and random forest regression (RF) models were employed, respectively, in the first stage and GRNN was used in the second stage. Two cases were considered to explore the performance of the FMG bands in estimating: (1) 3-DoF force and 3-DoF torque at once and (2) 6-DoF force and torque. In addition, the impact of sensor placement and the spatial coverage of FMG measurements were studied. This preliminary investigation demonstrates promising potential of FMG to estimate multi-DoF isometric force/torque. Specifically, *R*^2^ accuracies of 0.83 for the 3-DoF force, 0.84 for 3-DoF torque, and 0.77 for the combination of force and torque (6-DoF) regressions were obtained using the four bands on the arm in cross-trial evaluation.

## 1. Introduction

Hand force/torque estimation is essential for translating human intention into control commands to external devices for various applications including human-machine interaction (Merletti et al., 2004; Haddadin et al., 2008; Pervez and Ryu, 2008), laparoscopic surgery training (Hardon et al., 2018), prosthetic control (Nielsen et al., 2010), tele-operation (Khurshid et al., 2016) and tele-assessments of home-based rehabilitation (Zhang et al., 2016). For human-robot interaction, it is crucial for a machine to detect an unexpected increase of contact force when the operator tries to push the machine away to protect herself/himself from injury in any hazardous situations (Haddadin et al., 2008; Pervez and Ryu, 2008).

While traditional 6-degrees of freedom (DoF) force and torque sensors, such as the ATI Mini45 6-DoF force/torque transducer, could be used to detect force/torque exerted by a person to an object, they are generally expensive, bulky, and have to be attached directly to the object, which is not applicable in movable situations where pervasive and non-intrusive measurement of hand force/torque is critically needed. A potential alternative is to use lightweight and inexpensive sensors on the human upper limbs to measure the muscles contraction to estimate the exerted force/torque (Hof and Van den Berg, 1981; Cholewicki and McGill, 1994). Hand force/torque is generated by the activation or contraction of the corresponding muscles on the arm, and is a function of the Muscle Activation Level (MAL) (Mills, 2005). Thus, muscular signals generated from the activated motor units can be picked up at the surface of the skin in the vicinity of the electrode. Therefore, muscular sensing techniques such as the surface electromyography (sEMG) can be used to estimate hand force/torque (Mobasser and Hashtrudi-Zaad, 2005; Yang et al., 2009; Kamavuako et al., 2013).

Surface electromyography (sEMG) is frequently relied upon to provide non-invasive data of muscle activity. However, there has been recent interest in the development of alternative technologies to estimate hand movements and forces such as Force Myography (FMG). The advantages of FMG include that it does not require: (1) precise sensor placement regarding muscle anatomy, (2) extensive skin preparation, and (3) the same level of signal processing required in EMG datasets (Castellini and Ravindra, 2014). To aquire the FMG signals, force-sensing resistors (FSRs) are usually employed in a row/array that can be worn around the forearm or wrist (Ravindra and Castellini, 2014; Xiao and Menon, 2014). An FSR is a polymer thick film (PTF) which exhibits decreasing resistance with increasing applied force to the active area. As the hand exerts force or torque, the corresponding muscles located on the arm produce deformation on the surface of the skin. These deformations apply pressure to the surface of an FSR, and thus changing its resistance. These changes in resistance can be translated into corresponding changes in voltage that are digitized into the FSRs signals. Different hand gestures or variations in force/torque can result in distinct signal patterns that can be used for hand gestures or force/torque estimation.

To the best of the author's knowledge, there is no published study that simultaneously estimates multiple-DoF force or torque using FMG technology. Most of the current studies either using sEMG or FMG only estimate forces in 1-DoF, e.g., finger flexion-extension (Yang et al., 2009) or wrist pronation-supination (Ison et al., 2015). However, in a real situation, the wrist can perform multiple-DoF movements simultaneously, including flexion-extension, pronation-supination, and radial-ulnar. Sakr and Menon (2016) explored the feasibility of using FMG to estimate 3-axis wrist torque including: pronation-supination, radial-ulnar, and flexion-extension, by collecting each direction separately. Data were collected using a 1-DoF load cell where the participants exerted torque in one axis at a time, generating three FMG data sets. Then, the three separate 1-DoF FMG data sets were concatenated to train a model to predict the 3-axis torques. However, this is not a real 3-DoF torque estimation, since in a real situation, there are usually force/torque values distributed in more than one degree at a time even the participants intentionally focus on a single force/torque axis at a time.

Even using the more established sEMG technology, there are only a few published studies exploring multiple-DoF wrist force/torque sensing. Shahmoradi et al. (2015) proposed a method for estimating 3-DoF wrist force from sEMG acquired from the upper limb, for prosthetic control for trans-radial amputees. The authors first classified the sEMG signals into three classes (force axes) and then applied a neural network regression to associate the classified sEMG signals to corresponding wrist forces. The *R*^2^ accuracy of each DoF regression was reported to be high at around 0.92. However, the result was based on the randomized 5-fold cross validation for the second stage regression, where the training and testing data are mixed in time sequence and is not practical in a real use situation. Furthermore, the error introduced by the first stage classification would reduce the overall accuracy. Jiang et al. (2008) proposed a generative model called non-negative matrix factorization (NMF) to estimate 3-DoF wrist torque (flexion-extension, radial-ulnar, and pronation-supination) from sEMG. They achieved an *R*^2^ accuracy of 0.78 for 2-DoF force estimation (excluding the pronation-supination), but a poor accuracy was obtained for all 3-DoF force estimation together.

In this study, we explored the feasibility of using the FMG signals to simultaneously estimate 6-DoF hand force and torque exerted around the wrist joint. We recruited nine healthy participants to perform a sequence of six isometric wrist movements and a free exertion of isometric force and torque combined while wearing an array of 60 FSRs in four bands on the arm to collect the FMG data. A 6-DoF load cell was used to record the wrist force/torque to be used as the ground-truth data. The data was recorded while the participants maintain their forearm in the same position for all force and torque exertion sessions. A two-stage regression strategy was employed to enhance the performance of the FMG bands: in the first stage, the FMG signals were fed into a regression model to derive the 6-DoF output which was then used as input for the second stage regression. By employing the two-stage regression, the mutual information between different DoF signals was utilized to improve the accuracy. Three regression algorithms including general regression neural network (GRNN), support vector regression (SVR), and random forest regression (RF) models were employed, respectively, in the first stage and GRNN was used in the second stage. Two cases were considered to explore the performance of the FMG bands in estimating force/torque in: (1) 3-DoF force and 3-DoF torque combinations and (2) 6-DoF force and torque space. In addition, the impact of sensors placement and the spatial coverage of FMG measurements were studied. Sakr and Menon (2017) was a preliminary investigation for the best placement of the FMG bands on the arm for 3-DoF hand force prediction. In this paper, an extensive study for the best placement within four landmarks on the arm for FMG bands for improving the accuracy of 3-DoF force and torque prediction and 6-DoF force/torque prediction was provided.

The findings from the present study would expand the existing knowledge of using FMG for 1-DoF to multiple-DoF force/torque estimation. In addition, it provides guidelines for the research community about the best placement of the FMG bands for better force/torque sensing that can be combined with high density sensors technology (Koiva et al., 2015; Rasouli et al., 2015; Castellini et al., 2018) for the optimal sensing of hand force/torque in multi-DoF.

## 2. Proposed System and Experimental Setup

The system for data collection was composed of two parts. The first one was the force-sensing bands that capture the muscle contractions resulting from exerting force/torque. The other part was the custom rig that has a 6-DoF force/torque load cell built inside to collect the true values of the exerted force/torque.

### 2.1. Force-Sensing Band

Four customized force-sensing bands were designed to record FMG signals from the participant's working arm. Each band contains 16 force sensing resistors (FSRs, Model 402 from Interlink Electronics), except the wrist band which has 12 FSRs. The FSRs were arranged in series in each band and spaced 2 cm apart from each other. Snaps were placed on either side of the force-sensing band to allow the band to be securely donned. The FMG signals were digitized from the FSRs using a voltage divider circuit with a 4.7 k resistor that controls the sensitivity of the FSR and input voltage of 3.7 V. An ATMega328 microprocessor was used to facilitate the data collection and transmission. The FSRs were sampled at 10 Hz and the raw values were timestamped and transmitted to an on-site computer via a Bluetooth connection and saved in a file for offline processing. As the frequency of human hand motion is typically < 4.5 Hz, the 10 Hz sampling rate is sufficient for the purposes of this study (Xiong and Quek, 2006; Cho et al., 2016).

**Figure 1:**
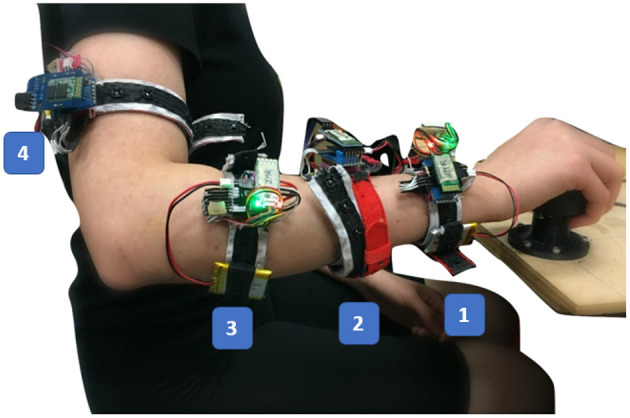
The participant holds the plastic sphere that accommodates the 6-DoF load cell during the data collection. The shoulder joint abduction angle is about 45° and the elbow angle is approximately 90°. The four bands positions are labeled as (1) the wrist band, (2) the midway band, (3) the muscle belly band, and (4) the upper arm band.

Toward the understanding of the effect of sensor placement and spatial coverage of FMG on the arm to the hand force/torque sensing around the wrist joint, four bands were simultaneously donned on the participants' arm while they completed a predefined protocol. The FMG bands were placed at the following four positions on the arm, respectively: (1) approximately 2.3 cm proximal to the wrist, identified by the surface land-marks of the radial and ulnar styloid processes (2) mid-way between the band at position 1 and the point on the forearm with the widest circumference (3) the point on the forearm with the widest circumference, and (4) the upper arm about 2 inches above the elbow. While the widest part of the forearm is characteristically associated with the muscle bellies of intrinsic forearm's musculature, for the purpose of results reporting, this land-mark is referred to as “the muscle belly of the forearm.” The placement of these four bands used for all the participants is shown in Figure 1.

### 2.2. 6-DoF Force/Torque Acquisition

**Figure 2:**
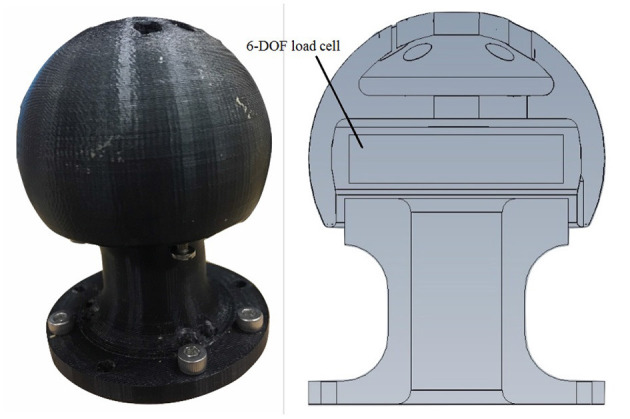
The 6-DoF force and torque data acquisition system. The left image shows the plastic sphere that houses the 6-DoF load cell and the right image shows a cross section sketch of the rig.

A custom-built rig was designed to measure the isometric hand forces and torques exerted onto the load cell in X, Y and Z axes, denoted as *F*_*X*_, *F*_*Y*_, *F*_*Z*_, *T*_*X*_, *T*_*Y*_, and *T*_*Z*_, respectively, in the rest of the paper. Figure 2 shows the custom-rig used for collecting the exerted force/torque. It is composed of a base that holds a hollow plastic sphere accommodating an ATI Mini45 6-DoF force/torque transducer. The resolution of *F*_*X*_, *F*_*Y*_, and *F*_*Z*_ is $\frac{1}{8}$ N and the resolution of *T*_*X*_ and *T*_*Y*_ is $\frac{1}{376}$ Nm and *T*_*Z*_ is $\frac{1}{752}$ Nm, respectively. The surface of the sphere was made rough to prevent hand slippage during the experiments. The transducer was connected to an interface power supply box to power it, as well as conditioning its signals to be used with a data acquisition system. The output of the interface power supply was connected to a data acquisition device (DAQ) from National Instruments (NI USB 6210).

A customized LabVIEW (© 2014) software was designed to collect both the applied force/torque from the transducer and the FMG signals from the FSR bands, with a visual chart showing the exerted force/torque values during the data collection. A laptop was used to run the LabVIEW software and connect to the FSR bands via Bluetooth and the transducer through a DAQ. The FMG and load cell data were synchronized and saved in a .CSV file for offline processing and analysis.

### 2.3. Experiment Protocol and Procedure

An experimental protocol was designed to collect the FMG signals and the exerted 6-DoF force/torque data for this study. Initially, each subject sat comfortably on a height-adjustable office chair, maintaining an upright position where the elbow angle was about 90° and the shoulder joint abduction angle was approximately 45°. The four bands were firmly and comfortably worn on specified spots on the participant's arm as in Figure 1. Then, the subject held the sphere on the custom-rig in his/her palm (Figures 1, 2) and started exerting isometric forces and torques in one of the 6-DoF with a predetermined order and duration. Each subject performed this protocol for five trials. In each trial, the subject tried to exert isometric forces in X, Y, and Z axes sequentially for 40 s for each axis while intended to keep the elbow and shoulder fixed in the initial position, the resultant force values are forming an approximate sinusoidal wave in each axis. Then, the participant tried to exert torques around the wrist joint: pronation-supination (*T*_*X*_), flexion-extension (*T*_*Y*_) and radial-ulnar (*T*_*Z*_) sequentially for 40 s for each axis. In the above session, the participant was instructed to exert force/torque each time in one axis, for the purpose of ease and to cover a relatively full range of each force/torque axis in the first 6 sessions of each trial. After that, the participant exerted both force and torque freely in a combination of different axes, we called this session “free-degree session.” During each trial, a visual chart was showed to display the exerted force/torque values with the resultant waves, to visually help the participant to maintain the sinusoidal wave form (except in the free-degree sessions) but without any limitations on the values or the speed of the force/torque exertion. The subjects were regularly checked whether they are feeling any fatigue in their arm muscles and they were allowed to rest their hands between the trials for few minutes if needed. At the end, there were 2,000 samples for each force/torque axis and the free-degree. During each trial, the FMG signals from the four bands and the six force/torque readings from the load cell were recorded and saved.

### 2.4. Participants

Nine healthy subjects with no known neuromuscular disorders (5 females and 4 males) aged (24 ± 2) participated in this study. The subjects were given a detailed oral description of the procedure. They affirmed their voluntary participation in an informed and written consent, and this protocol was approved by the Office of Research Ethics at Simon Fraser University.

## 3. Data Processing and Analysis

### 3.1. Data Processing

The data has been recorded using a total of 60 FSRs embedded in four bands. The number of sensors (active sensors) that were in touch with the participant skin during the data collection were manually recorded. In data processing, the inactive sensors data were removed. The number of active sensors varied from one participant to another based on the size of the participant's arm at the bands' positions. The average number of the active sensors used was 9, 10, 12, and 14 for band 1, band 2, band 3, and band 4, respectively.

Then, a high pass first order Butterworth filter with a cut-off frequency of 0.5 Hz was applied to remove the linear trend of each channel in the FMG signals. For both the true label and FMG signals, a low pass 5th order finite impulse response (FIR) filter with a cut-off frequency of 4 Hz was applied using Hamming window which was selected empirically.

After that, the five trials' data of each subject were divided into training and testing data, i.e., using one of the 5 trials as testing data and the remaining trials as training data, as described in section 3.3. Then the raw FMG signals were normalized using the minimum and maximum values of the training FMG data of each participant.

### 3.2. Two-Stage Regression Method

**Figure 3:**
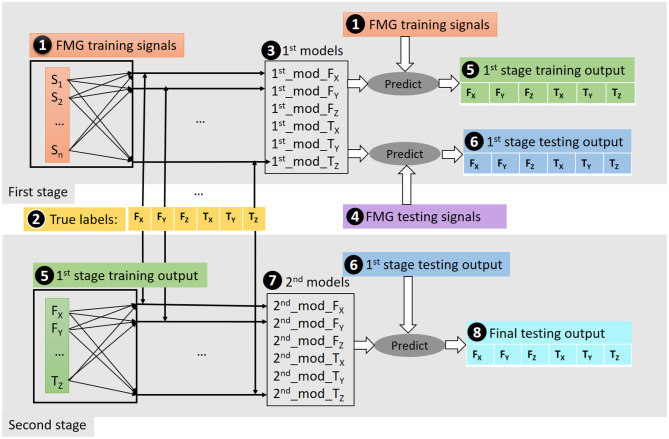
Illustration of the two-stage regression scheme. In the first stage, the FMG training data (➊) were paired with each of the six true labels (➋) to train six models (➌) for the 6-DoF force/torque estimation, respectively. Then these models were used to predict training (➎) and testing (➏) outputs based on the FMG training data (➊) and testing data (➍), respectively. In the second stage, as shown in the bottom panel of the figure, the estimated force/torque (➎1st stage training output) were used again together with the true labels (➋) to train six models (➐), which are then used to estimate final 6-DoF output (➑) from the testing output of the first stage (➏).

A two-stage regression processing was used with the preprocessed data, as shown in Figure 3. As stated in section 3.1, the FMG data were preprocessed and separated into training and testing data. The motivation for using two-stage regression is to capture the correlation among both the FMG signals and the exerted force/torque, and among the multi-axis force/torque themselves. In the first stage, the FMG training data (➊) were paired with each of the six true labels (➋) to train six models (➌) for the 6-DoF force/torque estimation, respectively. Then these models were used to predict training (➎) and testing (➏) outputs using the FMG training (➊) and testing data as inputs to the models (➍), respectively. In the second stage, as shown in the bottom panel of the figure, the force/torque estimated from the training data (➎1st stage training output) were used again together with the true labels (➋) to train six models (➐), which were used to estimate the final 6-DoF output (➑) using the testing outputs of the first stage (➏).

In the first stage, the derivative of the FMG signals were also used as a part of (➊) and (➍), respectively. This derivative information captures the trend of FMG amplitude change.

The second stage regression was employed for the purpose of utilizing the mutual information between the multi-axis force and torque. This is essential because there is always more than 1-DoF active at a time even though the intention of the participant was to exert force/torque in a specific direction. This can be clearly shown in Figure 9 where *T*_*Y*_ has considerable values in the trial of focusing on *F*_*Z*_. Similarly, *F*_*Y*_ and *T*_*Y*_ have significant values in the trial of focusing on *T*_*X*_. In addition, the two-stage method added extra features to the FMG signals by utilizing the output from the first stage models. Thus, the total features for the second stage were the raw FMG data, their derivative, the predicted 6-DoF force/torque from the first stage, their derivative, and spherical coordinate system transformation. The spherical coordinate system transformation was used to transfer data in cardinal space to the spherical coordinate system, for example, the force in X, Y, and Z directions was transformed to the corresponding spherical coordinates.

### 3.3. Regression Algorithms

Three regression algorithms were employed to model the FMG signals to the 6-DoF force/torque. These regression algorithms were successfully employed in regression problems for processing bio-signals like FMG and sEMG in several applications such as detecting single-finger forces (Castellini and Ravindra, 2014), hand prothesis control (Adewuyi et al., 2016; Connan et al., 2016), and estimation of knee joint angle (Benbakhti et al., 2014), and are briefly introduced as follows:

#### 3.3.1. General Regression Neural Network (GRNN)

**Figure 4:**
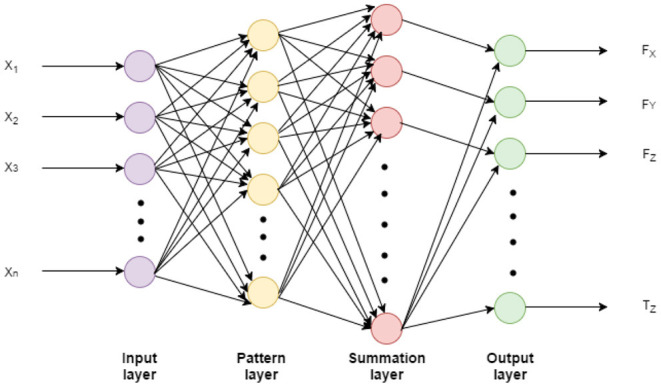
The GRNN architecture. The input *X*_1_,…, *X*_*n*_ are the FSRs channels, and the output *F*_*X*_,…, *T*_*Z*_ are the 6-DoF force/torque.

GRNN is able to handle multiple-output regression problems and has faster training speed than the typical back-propagation neural networks (Specht, 1991; Al-Mahasneh et al., 2018). Figure 4 demonstrates the structure of the GRNN used in this study. The GRNN network consists of four layers (Luh et al., 1999). First, the input layer has as many neurons as the number of input variables. Once the input goes through each unit in the pattern layer, the relationship between the input and the response would be recorded and stored in the unit. Thus, the number of units in the pattern layer is equal to the number of observations in the training sample. Then, the summation units perform a dot product between a weight vector and a vector composed of the signals from the pattern units. There are only two neurons in the summation layer for each output. One neuron is the denominator summation unit and the other is the numerator summation unit. The denominator summation unit adds up the weight values coming from each of the hidden neurons. The numerator summation unit adds up the weight values multiplied by the actual target value for each hidden neuron, as in (4). The addition of one element in the output vector requires only one summation neuron and one output neuron.

(1) $$\hat{Y}(x)=\frac{\sum_{i=1}^{n}Y_{i}\exp\left[\frac{-D_{i}^{2}}{2\sigma^{2}}\right]}{\sum_{i=1}^{n}\exp\left[\frac{-D_{i}^{2}}{2\sigma^{2}}\right]}$$

where $D_{i}^{2}={\left(X-X_{i}\right)}^{T}.\left(X-X_{i}\right)$

Where *X* is the input sample and *X*_*i*_ is the training sample memorized in the unit in the pattern layer. The output of the input sample *X*_*i*_ is *Y*_*i*_. $D_{i}^{2}$ is the Euclidean distance from the *X* and *X*_*i*_. It signifies how much the training samples can contribute to the estimated output of that particular test sample. If the distance $D_{i}^{2}$ is small, then the exponential in (4) will be a large value which means that the training sample will contribute more to the output estimation and vice versa. While if $D_{i}^{2}$ is zero, then the exponential returns one which means that the estimated output is the same as the training sample output. Finally, σ is the only unknown parameter which called spread constant. It was tuned in the training process to get the optimum value where the error is very small.

#### 3.3.2. Support Vector Regression (SVR)

Support Vector Regression (SVR) is one of the Support Vector Machine (SVM) techniques which is used for handling regression problems. SVR maps the input data to a higher-dimensional feature space where the data can be separated using the linear regression (Drucker et al., 1997). SVR does not suffer from the local minim problem since model parameter estimation involves solving a convex optimization problem (Bishop, 2006). Nu Support Vector Regression (ν-SVR) was used, as the ν parameter used to control the number of support vectors in the resulting model. We used the Radial Basis Function (RBF) kernel as in (5) it enables nonlinear mapping for the input data. Besides it has a small number of hyper parameters, which reduces model selection complexity (Wang et al., 2003).

(2) $$\begin{array}{l} k\left(x,y\right)=e^{-\frac{∥x-y{∥}^{2}}{2\sigma^{2}}} \end{array}$$

Where ∥*x* − *y*∥^2^ is the squared Euclidean distance between the two feature vectors and σ is the bandwidth of the RBF function.

#### 3.3.3. Random Forest Regression (RF)

Random Forest is a technique that can be used in regression and classification problems. It was introduced by Breiman (2001). The main idea is to train several decision trees, constituting a “Forest,” using a random sample of the dataset. After that, each tree is used independently to predict the output of a new data point. The final output of the whole forest is the combination of all these predictions, for example by averaging all of them. By using decision trees, a complex problem is splitted into smaller ones, which can be tackled efficiently using simple predictors.

The Random Forest algorithm (Friedman et al., 2001) starts by randomly drawing a sample of the training data before building the tree. To grow a random-forest tree, a subset of the input features is selected randomly. Then, for each terminal node of the tree, a split point among the training data is chosen such that the information gain is maximized. This node is then splitted into two children nodes. These steps are done recursively until a minimum node size is reached. The output is a Forest of ensemble trees ${\left\{T_{b}\right\}}_{1}^{B}$, where *T*_*b*_ is the tree number b and B is the total number of trees in the forest. In regression problems (like the case in this thesis), when a new point x is fed to the random forest, the predicted output *f*(*x*) is calculated according to (6):

(3) $$\begin{array}{l} f\left(x\right)=\frac{1}{B}\overset{B}{\underset{b=1}{\sum }}T_{b}\left(x\right) \end{array}$$

Criminisi et al. (2010) studied the effects of several parameters on the performance of the random forests. For example, they found that underfitting is likely to happen when the tree depth is small. In addition, increasing the depth may lead to the problem of overfitting. They also showed that as the forest size becomes larger, the decision boundaries reached by the random forest becomes better and smoother. When to comes to classification problems, the number of classes has almost no effect on the performance of the random forest.

Ten-fold cross validation on the training data and grid search were used to find the optimal values for the model parameters for the three algorithms, where the average accuracy of the 10 iterations is considered as the metric to compare between the different values of the algorithm's parameters, as it is usually done in common practice (Refaeilzadeh et al., 2007). This was done with every 4 training trials in the 5-fold cross-trial evaluation.

### 3.4. Data Analysis

The regression models were initially trained with the training data set using the two-stage process as described in section 3.2. The performance of the trained models was tested on the testing data set, by subsequently comparing the estimated force/torque values in 6-DoF with the true force/torque values recorded by the 6-DoF load cell, respectively. To quantify the performance of the resultant model, the coefficient of determination (*R*^2^) was employed as the performance metric. (*R*^2^) indicates how well the model fits the data. It was calculated as in (7), *R*^2^ with a value of 1 indicating the model perfectly fits the data and *R*^2^ of 0 completely does not fit the data.

(4) $$\begin{array}{l} R_{i}^{2}=1-\frac{\sum_{n=1}^{N}{\left({ŷ}_{i}\left(n\right)-y_{i}\left(n\right)\right)}^{2}}{\sum_{n=1}^{N}{\left(y_{i}\left(n\right)-\bar{y_{i}\left(n\right)}\right)}^{2}} \end{array}$$

where N is the number of data points, *y*_*i*_(*n*) is the true value of the ith force/torque, $\hat{y_{i}}$ is the corresponding estimate value and $\bar{y_{i}\left(n\right)}$ is the mean of the *i*th force/torque sequence over N data points.

Two cases were considered to study the suitability of using FMG signals to predict multi-DoF force/torque. The first case was involving building a FMG-based model to predict 3-DoF force and another one to predict 3-DoF torque. The second case was more extensive where a single model was used to predict the 6-DoF force/torque simultaneously. In each case, the regression model composed of two stage: the first stage used SVR, RF, and GRNN, respectively and the second stage used GRNN. We have tried all the three algorithms in the second stage and we found that there is no significant difference between them. For simplicity, we reported the GRNN results only in the paper. In addition, there are two reasons to use GRNN in the second stage regardless of different algorithms in the first stage. First, GRNN has a fast training speed (Specht, 1991) which is important to the two-stage model to decrease the required computations. Second, GRNN is able to handle multiple-output regression problems (Specht, 1991). This means that it needs only a single model to be trained to predict all the six outputs which simplifies the whole two-stage model.

The performance was calculated using the predicted force/torque values from the second stage compared to the true labels. We used cross-trial evaluation scheme in the two cases, where one trial within each subject was left for testing while the remaining four trials were used to train the regression model, this was done five times where in each time the testing trial was different, then the average accuracy was calculated across the five trials. This is motivated by studying the data consistency across different trials and also it is closer to the real situation. A two-way ANOVA was conducted to examine the effect of the two independent variables (the regression algorithms (applied in the first stage) and the FMG band combinations) to the dependent variable (the average *R*^2^ of second stage force/torque estimation), for the two cases. *Post-hoc* pair comparison (Tukey HSD) was further conducted if there was any significant effect of the variables on the accuracy. The significance level was set to α = 0.05.

## 4. Results

The data were successfully collected from all nine subjects each for five trials, except the last trial of subject 2, which was excluded due to the band slipping during the data collection. Thus, there were a total of 14,000 data points for each subject (five trials times six specific axes and one free 6-DoF sessions, 400 samples on each session). For subject 2, the data included only 12,000 samples and thus the cross-trial test was conducted in 4-fold and the average accuracy was from only four trials.

**Figure 5:**
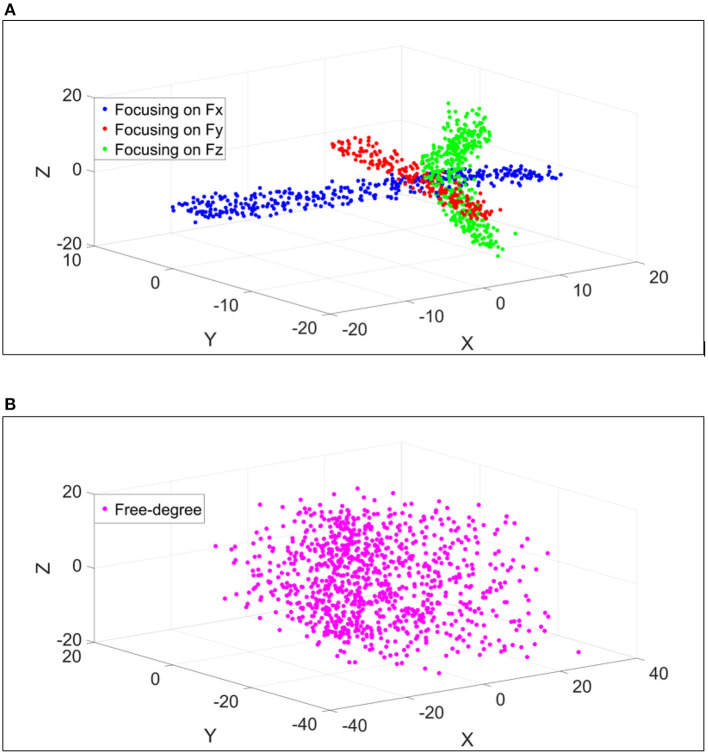
An example of the force values in X, Y, and Z that a participant exerted in few trials. The coordinates of each point are the force values in N in 3-DoF. **(A)** Represents the trials at which the participant focused on exerting force in one axis at a time. **(B)** Represents the trials at which the participant exerted force freely in all axes.

Figure 5A shows an example of data from a subject during trials where the participant focused on exerting force in one axis X, Y, or Z at a time. The force values in X, Y, and Z axes were considered as the coordinates of the points in the figure. It is shown that the points from the trial of focusing on one axis (e.g., X-axis) has the largest value in X-axis while the remaining axes (Y and Z axes) have small values. It is shown that the forces in three axes always active even though the intention is to focus on one axis at a time, but with the highest value in the axis which was intentionally desired and small values in the other axes. This justifies why the force data points in the trial that focus on X-axis are around the X-axis and the same observation for the other axes. Figure 5B represents an example of the free-degree trials, where the participant exerted force/torque freely in all axes.

It is noticeable that the data points are distributed among all three axes as the participant did not focus on a specific axis.

Since the applications in the real-world may require various types of limb force/torque monitoring in terms of estimating one axis at a time or estimating combined axes, we designed the protocol to cover these needs where there were sessions that focused on one axis at a time and the others were covering the combined axes. In addition, this design allows to have sufficient data points from each axis to train the model to estimate accurately. Also, we choose the sinusoidal wave to be the pattern of the exerted forces/toques to cover a relatively full range of each force/torque axis.

**Figure 6:**
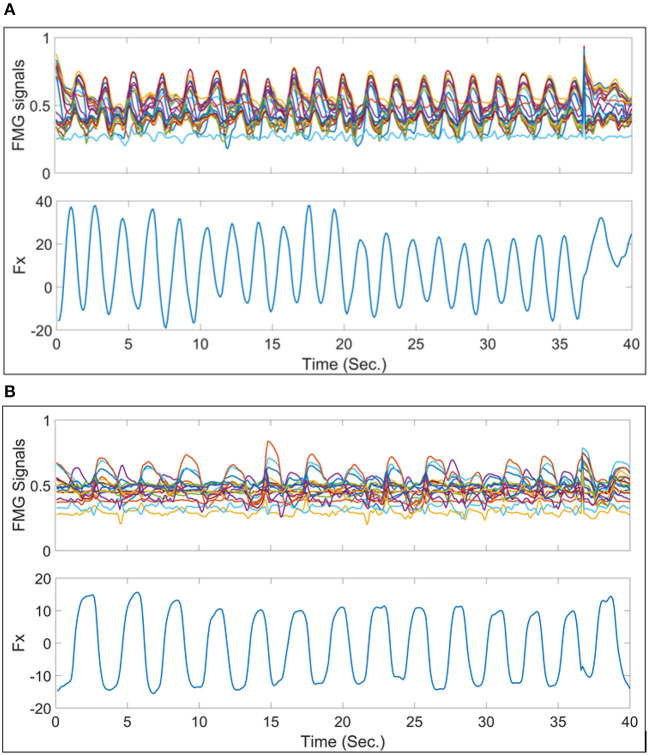
Samples of the normalized FMG signals from band 3 vs. the force exerted in X-axis in one trial for 40 s. **(A)** Presents one of the best signals where the FMG signals follow the same pattern as the force exerted in X-axis. **(B)** Presents one of the worst examples of FMG signals vs. *F*_*X*_ signals.

Figure 6A shows a sample trial of the normalized FMG signals from band 3 data vs. the exerted force in X-axis (*F*_*x*_). It is shown that the FMG signals have a correlated pattern like that of *F*_*x*_. As mentioned in section 3.4 there is two cases were considered to study the viability of using FMG signals to predict multi-output force/torque. In the following subsections, the accuracies of the regression models is presented for these two cases.

### 4.1. Case 1: Two Models for 3-DoF Force and 3-DoF Torque Regression Respectively

The first study explored the accuracies of using FMG in estimating 3-DoF force and 3-DoF torque separately. One regression model was trained to estimate the force in X, Y, and Z axes, and another model was trained to estimate the torque around 3-axis: flexion-extension, pronation-supination, and radial-ulnar. The input to the force model was the FMG signals collected during the trials of force exertion in X, Y, and Z, and the input to the torque model was the FMG signals of the torque exertion trials.

**Figure 7:**
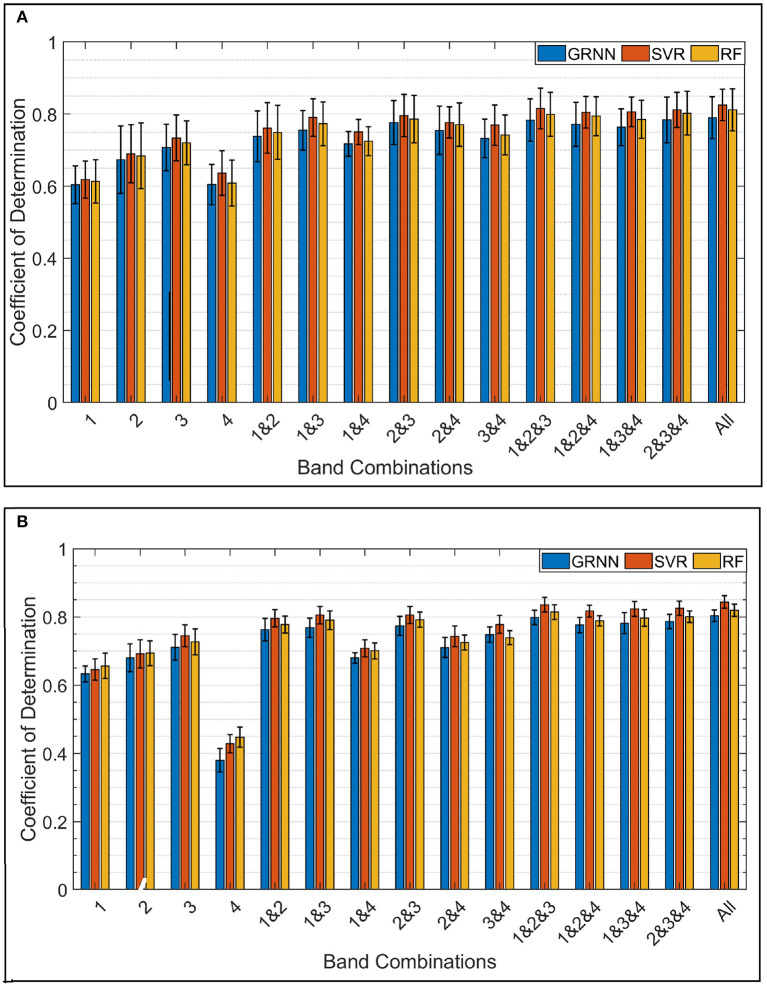
**(A)** Shows the average 3-DoF *R*^2^ across all subjects for force estimation in X, Y, and Z axes using GRNN, SVR, and RF, respectively, in the first regression stage and GRNN in the second stage. **(B)** Shows the average 3-DoF *R*^2^ across all subjects for torque estimation in X, Y, and Z axes using same regression algorithms.

Figure 7A shows the average *R*^2^ of the force in X, Y, and Z axes across all subjects using GRNN, SVR, and RF in the first stage and GRNN in the second stage regression. While Figure 7B shows the average *R*^2^ of the torque estimation around X, Y, and Z axes across all subjects. The accuracy was calculated by comparing the true labels vs. the predicted force/torque value from the second stage regression and averaging the resulting accuracies across the trials and DoFs. It is shown that all regression algorithms have comparable estimation accuracies for both 3-DoF force and torque. In addition, it is clearly shown that increasing the number of FMG bands used, increased estimation accuracy and decreased the standard deviation across subjects.

Using all bands achieved the highest average *R*^2^ for 3-DoF force estimation with 0.79 ± 0.06, 0.83 ± 0.04, and 0.81 ± 0.06 using GRNN, SVR, and RF, respectively. Similarly, for 3-DoF torque estimation with average accuracies of 0.80 ± 0.02, 0.84 ± 0.02, and 0.82 ± 0.02 respectively, using the same algorithms. While decreasing the number of FMG bands to 3, the accuracy was slightly decreased or remained steady. The combination of bands 1, 2, and 3 (on the wrist, forearm midway and forearm muscle belly) achieved the highest accuracy among all triple-band combinations for both force and torque estimation. The average accuracies using combination of bands 1, 2, and 3 for 3-DoF force estimation were 0.78 ± 0.06, 0.82 ± 0.06, and 0.80 ± 0.06, and for 3-DoF torque estimation were 0.80 ± 0.02, 0.84 ± 0.02, and 0.81 ± 0.02 using GRNN, SVR, and RF, respectively.

For double-band combinations, the combination of bands 2 and 3 achieved the highest accuracy among all double-band for both 3-DoF force and torque estimation. The accuracies for force estimation were 0.78 ± 0.06, 0.80 ± 0.06, and 0.79 ± 0.07 using GRNN, SVR, and RF, respectively. While torque estimation accuracy using the same bands combination were 0.77 ± 0.03, 0.81 ± 0.02, and 0.79 ± 0.02 using the three regression algorithms, respectively.

In the more challenging situation of using single FMG band to estimate multi-DoF force/torque, a moderate accuracy for both 3-DoF force and torque estimation was still achieved. Among the four placement positions used, the band on the forearm muscle belly (band 3) achieved the highest accuracy. In this situation, the average accuracies for force estimation were 0.71 ± 0.06, 0.73 ± 0.06, and 0.72 ± 0.06, using GRNN, SVR, and RF, respectively. Similarly, torque estimation accuracies were 0.71 ± 0.04, 0.75 ± 0.03, and 0.73 ± 0.04 using the same regression algorithms, respectively.

The two-way ANOVA showed significant effects of the band combinations [*F*_(14, 404)_ = 18.1, *p* < 0.00001] and regression algorithm [*F*_(2, 404)_ = 3.54, *p* < 0.05] to the mean *R*^2^ of hand force/torque estimation; there was no significant interaction effect of the band combinations and regression algorithms. The *Post-hoc* test (Tukey HSD) on the effect of band combinations showed that among the 4 single band positions, band 3 achieved significantly higher *R*^2^ compared to band 1 (*p* < 0.05) and band 4 (*p* < 0.00001), and band 4 had the lowest accuracy compared to other 3 single bands (*p* < 0.01). There was no significant difference between the accuracies of using single-band band 3 and the combinations involving multiple bands. As expected, there was also no significant difference of *R*^2^ between using any of the double-band combinations, triple- and quad-bands, except the double-band combination (bands 1 and 4) has significantly lower accuracy than those of using triple-band 1, 2, and 3 (*p* < 0.05) and all 4 bands (*p* < 0.05). The *Post-hoc* test on the effect of algorithm showed that the *R*^2^ of using GRNN was significantly lower than that of SVR (*p* < 0.05). In addition, there was no significant difference between either RF and GRNN or RF and SVR.

### 4.2. Case 2: One Model for all 6-DoF Force and Torque

**Figure 8:**
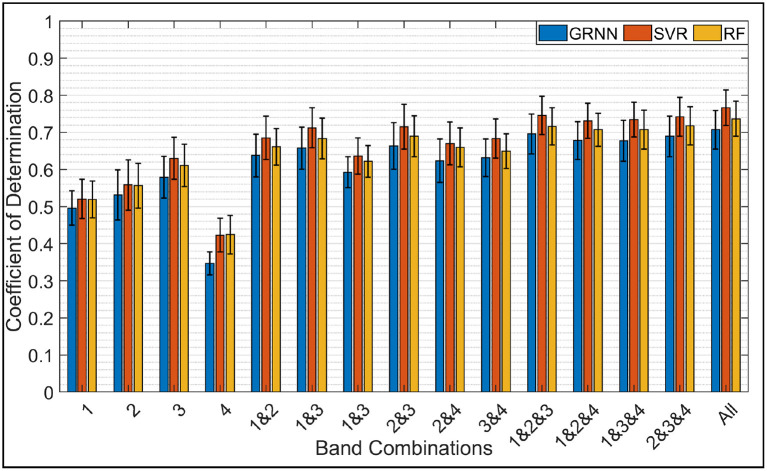
Average *R*^2^ across all subjects for the force/torque estimation in 6-DoF using GRNN, SVR, and RF.

The second case was more challenging where only one FMG-based model was used to estimate the force/torque in 6-DoF. Figure 8 demonstrates the average estimation accuracies (6-DoF *R*^2^) across all subjects using GRNN, SVR, and RF in the first stage and GRNN in the second stage. Similar to 3-DoF force/torque results, all regression algorithms performed with comparable accuracy. In addition, it is clearly shown that the accuracy significantly surged when using all bands compared to single band. The accuracy using all bands almost double the accuracy using band 4 only.

Using all bands achieved the highest accuracy among all band combinations. The average 6-DoF *R*^2^ were 0.71 ± 0.05, 0.77 ± 0.05, and 0.74 ± 0.05 using GRNN, SVR, and RF, respectively. Using triple-band combination, the accuracies were slightly decreased. Among all triple-band combinations, the combination of bands 1, 2, and 3 achieved the highest accuracy of 0.70 ± 0.05, 0.75 ± 0.05, and 0.72 ± 0.05.

Decreasing the spatial coverage of the used FMG bands to double-band combinations, the accuracy was decreased by 0.04 on average compared to using all 4 bands. Similar to 3-DoF force and torque results, combination of bands 2 and 3 achieved the highest accuracies among all double-band combinations with an average accuracy of 0.66 ± 0.06, 0.72 ± 0.06, and 0.69 ± 0.06 using GRNN, SVR, and RF, respectively. Using single-band, band 3 also has the highest accuracy with an average *R*^2^ of 0.58 ± 0.06, 0.63 ± 0.06, and 0.61 ± 0.06 using GRNN, SVR, and RF, respectively.

The two-way ANOVA showed both significant effects of the band combinations [*F*_(14, 404)_ = 28.48, *p* < 0.00001] and regression algorithm [*F*_(2, 404)_ = 10.44, *p* < 0.00001] to the mean *R*^2^ of hand force/torque estimation; there was no significant interaction effect of the band combinations and regression algorithms. The *Post-hoc* test (Tukey HSD) on the effect of band combinations showed that among the 4 single band positions, band 3 achieved significantly higher *R*^2^ compared to band 1 (*p* < 0.0005) and band 4 (*p* < 0.00001), and band 4 had the lowest accuracy compared to other 3 single bands (*p* < 0.00001). The accuracies of using any of the three single-band in position 1, 2, and 4 were significant lower compared to any of the band combinations involved more than single band (*p* < 0.05). However, the *R*^2^ of band 3 was only significantly lower than those of using two of the double-band combinations: bands 1 and 3 (*p* < 0.05), bands 2 and 3 (*p* < 0.005), all triple-band combinations (*p* < 0.00001), and all 4 bands together (*p* < 0.0005). Among the 6 double-band combinations, only the combination bands 2 and 3 achieved a significant higher accuracy than the combination of bands 1 and 4 (*p* < 0.05), but there was no significant difference of *R*^2^ between using any of the other double-band combinations. Interestingly, there was no significant difference of *R*^2^ between the double-band combination bands 2 and 3 and any of the combinations of triple- and quad-band. That is using only 2 bands on the forearm is able to achieve comparable accuracy as using 3 and 4 bands.

The *Post-hoc* test (Tukey HSD) on the effect of regression algorithms showed that the *R*^2^ of using GRNN was significantly lower than those of SVR (*p* < 0.00001) and RF (*p* < 0.05). There was no significant difference between SVR and RF.

It was also interesting to see the improvements in the regression accuracy using two-stage model compared to single-stage. An ANOVA analysis was performed to compare between the 6-DoF accuracy using all bands with single-stage model vs. two-stage model. The accuracies (*R*^2^) have been significantly improved (*F* = 53.37, *p* < 0.0001), from 0.69, 0.74, and 0.71 to 0.71, 0.77, and 0.74 using GRNN, SVR, and RF, respectively.

**Figure 9:**
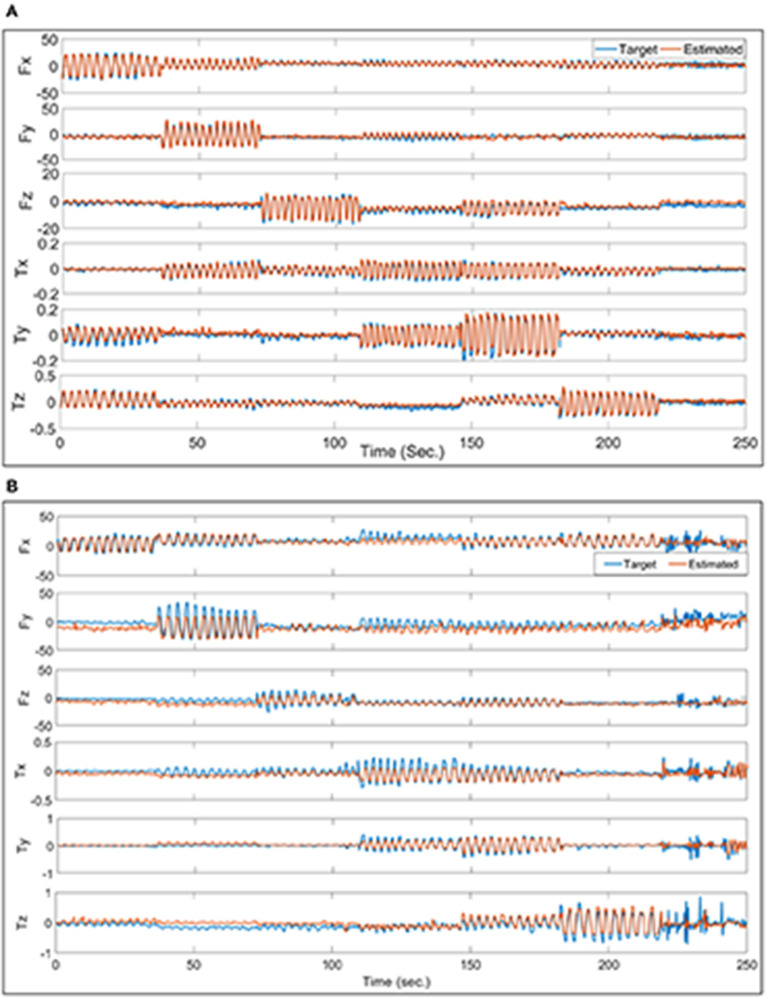
**(A)** Is an example of the best performance on the force/torque estimation for one trial from the data of subject 3 where all 4 bands were used. **(B)** Is a sample of the worst performance. The forces in both charts are in N while the torques are in Nm.

Figure 9A shows an example of a good estimation for the force and torque in 6-DoF using all 4 bands from testing one trial from the data of subject 3. The 6-DoF *R*^2^ for this trial is 0.90. On contrary, Figure 9B shows an example of a poor estimation with 6-DoF *R*^2^ of 0.68 from testing the model with the third trial of the subject 4 data. In addition, Figure 9 shows the order of exerting force/torque on each axis specifically, and in the last part of each chart shows the free-degree as the participant exerts force/torque freely in any axis.

## 5. Discussion

The results showed the viability of employing FMG signals to estimate multiple-axis hand force/torque using regression models. The FMG on the arm achieves acceptable accuracies of 0.83 and 0.84 in 3-axis force and torque combinations and 0.77 in 6-DoF force/torque regression, averaged across nine participants using the four bands on the arm. The result from the present study is promising and has implications for various applications. For instance, the FMG based technology could be employed in human robot collision detection (Haddadin et al., 2008), which will enable a ubiquitous and low-cost solution for HRI safety through force/torque monitoring. In tele-assessments of home-based rehabilitation, this FMG based wearable 6-DoF force/torque sensing technology offers potential to simultaneous multiple-DoF force/torque estimation instead of only using a downward axis force estimation (Zhang et al., 2016).

In this study, a sophisticated two-stage regression model was proposed to enhance the estimation performance by utilizing the mutual information between force/torque axes. Three major-stream machine learning algorithms were explored in the first stage regression of the model.

Another interesting finding is that the best placement of the FMG band among the proposed four landmarks was the forearm muscle belly. This finding suggests that the primary location for isometric hand force/torque sensing should be the forearm muscle belly, and the upper arm is not suitable for wrist torque sensing. This finding also sheds light on the design of the FMG band in real applications, i.e., the sensor density should be increased along the forearm widest part toward the lower forearm to effectively enhance the performance. Knowledge gained from this study can be applied to human-robot interaction safety assessment, design, and planning and control (Pervez and Ryu, 2008).

The characteristics and categories of the muscles during isometric hand force/torque exertion can help to explain the above phenomenon. First, band 3 achieved the highest accuracy because the muscles on the forearm muscle belly including anconeus, branchioradialis and pronator teres contribute actively to all the 6 axes isometric force/torque generation, due to the resistance from the object that the subject is pushing against (Buchanan et al., 1986). Secondly, the exertion of the hand forces *F*_*X*_, *F*_*Y*_, and *F*_*Z*_ also involves the activation of the muscles on the upper arm, including long bicep brachii, short head bicep brachii, and brachialis muscles (Buchanan et al., 1989). For instance, to exert hand force in the X axis, the participant might try to rotate the upper arm around the shoulder joint, and/or adduct/abduct the shoulder. Lastly, the wrist torques (*T*_*X*_, *T*_*Y*_, and *T*_*Z*_) are mainly resulted from the contraction of the muscle on the forearm, which results the low accuracies of the band 4 on the upper arm for torque sensing. The result *R*^2^ = 0.83 for 3-DoF force estimation achieved in this study is much higher than those of the studies using the well-established sEMG technology in estimating force for less dimensions, e.g., 2-DoF wrist torque estimations with *R*^2^ = 0.78 (Jiang et al., 2008). Even in the estimation of 6-DoF force/torque simultaneously in this study (which usually lead to increase the estimation difficulty), the results is of comparable *R*^2^ = 0.77.

Furthermore, during the data collection, we did not limit either the max/min values of the exerted force/torque or the speed of force/torque exertion, e.g., by using a predefined visual chart to guide the force/torque exertion. This relatively freely exerted force/torque would increase the inconsistency of the data between trials, which is usually more of a challenge to estimate compared to those using more constrained protocols (Jiang et al., 2008; Nielsen et al., 2010; Kamavuako et al., 2013). In addition, some subjects slightly rotated their elbow and/or adducted/abducted their shoulder during the data collection which made the data inconsistent between different trials and possibly affected the accuracy. Even with these challenges, the results from this study still show competitive accuracies. On the other side, although the relatively less-restricted experimental protocol poses challenges in signal processing and force/torque estimation and thus decreases the evaluation accuracy, there are practical values by doing this, as it reflects more to real application scenarios compared to those restricted ones (Jiang et al., 2008; Nielsen et al., 2010; Kamavuako et al., 2013). The fact is that in real application scenarios, it is hard and unpractical to ask the users to keep ideal consistency in force/torque exertion along time in terms of force/torque range and arm/elbow position. For instance, unexpected movements may happen during real applications.

There is a phenomenon that, although the participant intended to try exerting force/torque each time in one axis, the forces and torques recorded by the 6-DoF sensors show that there are usually more than one degree of force/torque active at a time, as shown in Figure 5. This phenomenon repeated during the rest segments, and was also reported in Kamavuako et al. (2013) and Formica et al. (2012). This also implies that there is a need to estimate multiple degrees of force/torque simultaneously while using sensing technologies such as FMG, even when the applications mostly rely on 1-DoF force/torque estimation at one time. Mostly the mis-regression happens at the turn-around points of these sinusoidal waves, where the force/torque axis changes as shown in Figure 9B. This might have referred to several reasons, as shown in Figure 6B the FMG signals from the forearm muscle belly band did not follow the sinusoidal pattern well compared to Figure 6A. In addition, there was a variance in the amplitude of the exerted force which affected the estimation accuracy as the training data did not finely represent the testing data.

This degradation in the accuracy along with reducing the number of FSR bands used affirms the previous finding in Radmand et al. (2016); that is, higher FSRs density increases the prediction ability, and suggests that only a single band on the arm might not be sufficient for the estimation of 6-DoF forces and torques.

## 6. Limitations and Future Work

This study was limited to a lab setting purposed to study the feasibility of using the prototype FMG bands to estimate the hand force/torque based on the data mainly from 6 axes force and torque plus a relatively limited data set of free 6-DoF force/torque exertions. Future studies should expand the free 6-DoF force/torque data collection to cover a sufficient amount of points in the 6-DoF space for the model training to improve the estimation performance of the band. The participants were asked to exert isometric force/torque that formulate sinusoidal waves with a visual feedback of the resultant pattern from the values of the exerted forces and torques. As several factors like the arm position and the type of movement (isometric or dynamic) could affect the estimation accuracy in a real case scenario, further exploration in situations more closely simulating a real-life setting with less constrain of arm/elbow joint angle should be conducted in the future study.

Based on the finding from this study that the position at the forearm muscle belly achieves the best performance, future work should design a high density FMG band that covers a large area of the forearm starting from the forearm muscle belly to further improve the regression accuracy. Notice that several issues have not been included in this paper, but are crucial when applying the FMG technology toward real scenarios, including reducing training effort of the users and increasing the robustness of the system with respect to the variation of the sensor set-up, such as calibration work after DoFfing and donning the band and generic model which is applicable between users. Our research group is making effort to address these problems and the possible findings will be published in the coming papers in the future.

## 7. Conclusion

The present study explored the viability of using the FMG on the arm for 6-axis hand force/torque estimation, and examined the effects of the FSRs density and location to the force/torque estimation accuracy. Nine subjects participated in this study by exerting isometric force/torque in 6 axes, while the FMG signals were recorded by 4 FMG bands worn on the arm and the forces and torques were recorded by a 6-DoF load cell to label the data. A two-stage regression strategy was employed to enhance the performance of the FMG bands, where three regression algorithms including support vector regression (SVR), general regression neural network (GRNN), and Random Forest (RF) models were employed, respectively, in the first stage and GRNN was used in the second stage. The resulting accuracies were tested by 2-way ANOVA to find out the best location on the arm for the hand force/torque estimation. The results showed that FMG achieves a good performance in multiple-DoF force/torque estimation, with an average *R*^2^ of 0.83 and 0.84 in 3-axis force and torque combinations, and 0.77 in 6-DoF force/torque regression were obtained using the four bands on the arm in cross-trial evaluation. In addition, the results of 2-way ANOVA showed that the location on the forearm muscle belly (band 3) was the best for isometric hand force/torque sensing using the FMG signals. The findings from this study confirm the viability of using the FMG signals from the arm for multi-axis isometric hand force and torque around the wrist estimation, and knowledge gained from this study will provide guidance for hand force/torque estimation in terms of optimal FSR sensors placement and density.

## Data Availability Statement

The datasets generated for this study are available on request to the corresponding author.

## Ethics Statement

The studies involving human participants were reviewed and approved by Office of Research Ethics at Simon Fraser University. The patients/participants provided their written informed consent to participate in this study.

## Author Contributions

CM was principal investigator of the research, conceptualized the study (grant: Automotive Partnership Canada). MS designed and implemented the user study to evaluate the system. MS collected the data and analyzed it with XJ. XJ performed statistical analysis. MS wrote most of the paper while XJ wrote some sections and revised the paper. All authors contributed to the manuscript revision, read, and approved the submitted version.

### Conflict of Interest

The Principal Investigator, CM, and members of his research team have a vested interest in commercializing the technology tested in this study, if it is proven to be successful and may benefit financially from its potential commercialization. The remaining authors declare that the research was conducted in the absence of any commercial or financial relationships that could be construed as a potential conflict of interest.
